# Galanin(1-15) Potentiates the Antidepressant-like Effects Induced by Escitalopram in a Rat Model of Depression

**DOI:** 10.3390/ijms221910848

**Published:** 2021-10-07

**Authors:** Laura García-Durán, Antonio Flores-Burgess, Noelia Cantero-García, Araceli Puigcerver, José Ángel Narváez, Kjell Fuxe, Luis Santín, Carmelo Millón, Zaida Díaz-Cabiale

**Affiliations:** 1 Faculty of Medicine, Institute of Biomedical Research of Malaga, Campus de Teatinos s/n, University of Malaga, 29071 Malaga, Spain; lauragd_24@uma.es (L.G.-D.); afburgess@uma.es (A.F.-B.); noeliacg@uma.es (N.C.-G.); bueno@uma.es (J.Á.N.); 2 Faculty of Psychology, Institute of Biomedical Research of Malaga, Campus de Teatinos s/n, University of Málaga, 29071 Malaga, Spain; araceli@uma.es (A.P.); luis@uma.es (L.S.); 3 Department of Neuroscience, Karolinska Institute, 17177 Stockholm, Sweden; Kjell.Fuxe@ki.se

**Keywords:** Galanin(1-15), Escitalopram, depression, olfactory bulbectomy rats

## Abstract

Selective 5-HT reuptake inhibitor antidepressants (SSRIs) are the first choice in major depressive disorder (MDD), but 50% of affected patients do not show improvement. Galanin(1-15) [GAL(1-15)] enhanced Fluoxetine antidepressant-like effects in an animal model of depression, the olfactory bulbectomy (OBX); however, further detailed analysis of GAL(1-15) effects as augmentation treatment in OBX rats are needed. In OBX rats, we analysed the effect of GAL(1–15) on Escitalopram (ESC)-mediated responses in behavioural tests related to despair. We studied whether GAL(1–15) effects involved 5-HT1AR using an in vivo model siRNA 5-HT1A knockdown rats. Moreover, we analysed by immunohistochemistry the expression of the immediate-early gene c-Fos (c-Fos IR) after the administration of GAL(1-15)+ESC in OBX rats in several nuclei involved in MDD. GAL(1-15) enhances the antidepressant-like effects of ESC, and the GALR2 antagonist M871 blocked GAL(1-15) mediated actions. The downregulation of 5-HT1AR by siRNA was sufficient to block GAL(1-15) effects. Our immunohistochemistry and principal component analysis (PCA) analysis suggest that two functional networks are involved in these effects; one includes the lateral (LHb) and medial (mHb) habenula, dorsal raphe (DR) and ventral tegmental area (VTA), and the other consists of the dentate gyrus (DG), and prefrontal cortex (PFC). The results open up the possibility of using GAL(1-15) in combination with SSRIs as a novel strategy for treating MDD.

## 1. Introduction

Depression or major depressive disorder (MDD) is one of the most disabling mental disorders. According to estimates by the World Health Organisation, it affects 350 million people and in 2030 it will become the leading cause of incapacity of the world [1,2].

This complex emotional disorder is often characterised by hopelessness, anhedonia, exacerbated guilt, and painful physical symptoms [3], resulting in suicidal thoughts and attempts [4]. Most pharmacological treatments for MDD act by modifying neurotransmission mediated by serotonin (5-HT) and norepinephrine to mitigate these symptoms. Selective 5-HT reuptake inhibitor antidepressants (SSRIs) are frequently used as the first choice because they are highly effective, well-tolerated, and have higher adherence rates than other drugs. However, 50% of affected patients do not show improvement after being treated with two or more drugs or psychotherapeutic interventions [5,6]. Therefore, there is a need to develop novel treatments that provide effective, faster and prolonged relief of depressive symptoms in patients with MDD.

In this context, neuropeptides and their receptors, the most diverse family of neurotransmitters in the brain, have been extensively explored, and the neuropeptide Galanin (GAL) is under extensive preclinical investigation [7,8].

GAL, upon its action on its three GAL receptors (GALR1-3) [9,10], participates in numerous physiological processes and different disease, including mood regulation and depression in animal models [11,12,13]. Thus, the activation of GALR1 and GALR3 results in depression-like behaviour, while stimulation of GALR2 leads to anti-depressant-like effects [14,15,16].

Notably, the pro-depressant effect of GALR1 is attributed to its ability to modulate auto-, and heteroreceptor 5-HT1A (5-HT1AR) functions through GALR1-5HT1A heteroreceptor complexes, especially in the limbic forebrain regions and in the dorsal raphe (DR) [17,18].

GAL and the N-terminal fragment GAL(1-15) are active in the central nervous system [19,20,21]. GAL(1-15) acting through GALR1-GALR2 heteroreceptor complexes, especially in the dorsal hippocampus and DR [18,22] results in depression and anxiogenic-like effects [21,22,23]. In addition, GAL(1–15) enhances the antidepressant effects induced by the 5-HT1AR agonist 8-OH-DPAT [23], which involves alterations in both the binding characteristics and mRNA levels of 5-HT1AR in the dorsal hippocampus and DR [23].

Thus, considering the possibility to use GAL(1-15) as a combined treatment with SSRIs to improve their effectiveness, we observed in rats that GAL(1-15) enhanced the antidepressant effects and reversed the memory impairment induced by Fluoxetine (FLX) being involved the 5-HT1AR in the hippocampus and prefrontal cortex (PFC), respectively [24,25].

Recently we have also observed an interaction between GAL(1-15) and FLX in an animal model of chronic depression, the olfactory bulbectomised rat (OBX) [26]. These animals mimic several symptoms observed in patients with MDD, including anhedonia in the sucrose preference test, increased hyperactivity in a new environment, reduced sexual activity, and elevated corticosterone levels [27].

In this model, GAL(1-15) also enhances the antidepressant-like effects induced by FLX in behavioural tests related to despair and anhedonic behaviour, one of the characteristic symptoms of MDD [26]. The mechanism underlying the GAL(1-15)/FLX interactions in the OBX animals involves the 5-HT1AR in the hippocampus at the plasma membrane and transcriptional levels. The analyses of the circulating levels of corticosterone after the combined administration of GAL(1-15) and FLX also indicate that only the combination of both compounds can reduce the high levels of corticosterone characteristics of these animals [26].

All these results suggest the possible use of GAL(1-15)+FLX as a treatment for several symptoms associated with depression. However, further detailed analysis of the effects of GAL(1-15) as an augmentation treatment with other SSRIs, the first-line pharmacotherapy for the treatment of MDD, and the areas involved in the effect are needed.

We have selected Escitalopram (ESC), a pure S-enantiomer 100 folds more potent than the R-enantiomer to inhibit 5-HT reuptake and with no or very low-affinity for other receptors [28]. In OBX rats, we have analysed the effect of GAL(1–15) on ESC-mediated responses in behavioural tests related to despair. Therefore, we studied whether GAL(1–15) effects on ESC actions involved GALR2 with the GALR2 antagonist M871 and via 5-HT1AR using an in vivo model siRNA 5-HT1A knockdown rats. Moreover, we have analysed by immunohistochemistry the expression of the immediate early gene c-Fos (c-Fos IR), an indirect marker of neural activity, after the administration of GAL(1-15)+ESC in OBX rats in several nuclei involved in MDD: Hippocampus, PFC, lateral (LHb) and medial (mHb) habenula. Double immunohistochemical staining of 5-hydroxytryptamine (5-HT) and c-Fos-immunoreactivity (IR) or tyrosine hydroxylase (TH) and c-Fos were used to study the specific cell activation in the DR and the ventral tegmental area (VTA), respectively, after GAL(1-15)+ESC. Additionally, we assessed the brain networks or circuits involved using principal component analysis (PCA), a multivariate analysis used to understand brain functional organisation.

## 2. Results

Fourteen days after the bilateral olfactory bulbectomy surgery, OBX rats displayed increased locomotor activity in the open-field showing an increase in the total distance (t_22_ = 2.56; *p* = 0.008) and velocity-time (t_22_ = 2.56; *p* = 0.008) (Supplementary Table S1). These results confirmed that bulbectomised rats displayed the expected behavioural changes.

Moreover, in the dorsal hippocampus OBX rats exhibited an increased in BDNF (t_9_ = 3.87; *p* = 0.001), TRKb (t_9_ = 5.58; *p* = 0.0002), Rab5 (t_9_ = 2.26; *p* = 0.025), and 5HT1A (t_9_ = 3.74; *p* = 0.002) expression (Supplementary Table S2). In the PFC OBX showed an increase in 5HT1A expression (t_9_ = 4.08; *p* = 0.001) and a decreased in Home1A expression (t_9_ = 3.47; *p* = 0.003), while the bilateral olfactory bulbectomy rats lacked effect in the mRNA expression levels of GALR1, GALR2 receptors (Supplementary Table S2).

### 2.1. Behavioural Effects

#### 2.1.1. In OBX Rats, GAL(1-15) Enhanced ESC-Effects in Two Behavioural Test Related to Behavioural Despair. GALR2 Antagonist M871 Blocked the Behavioural Effects of GAL(1-15) in the FST

**Figure 1:**
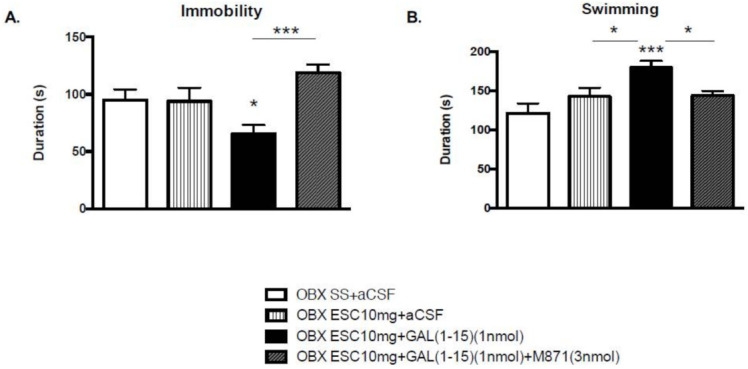
Behavioural effects of the coadministration of effective dose of ESC (10 mg/kg) and GAL(1-15) (1 nmol) in the forced swimming test (FST). Saline or ESC was administered intraperitoneal (ip) 23, 5, and 1 h before the test. aCSF, GAL(1-15) or M871 were injected intracerebroventricular (icv) 15 min before the test. Data represent mean ± SEM of immobility and swimming time in FST during the 5 min test period (n = 7–11 rats per group). In (**A**) * *p* < 0.05 versus OBX SS+aCSF and OBX ESC10mg+aCSF, in (**B**) *** *p* < 0.001 versus OBX SS+aCSF, according to one way ANOVA followed by Fisher multiple comparison test.

The threshold dose of GAL(1-15) (1 nmol) enhanced the antidepressant-like effects mediated by the three intraperitoneal (ip) injections of ESC (10 mg/kg) since the overall one way ANOVA revealed a reduction in the immobility time (one-way ANOVA, F_3,33_ = 5.20, *p* = 0.004) and an increase in the swimming time (one-way ANOVA, F_3,33_ = 6.30, *p* = 0.001) (Figure 1). Intracerebroventricular (icv) GAL(1-15) significantly decreased the immobility time induced by ESC (10 mg/kg) by 30% in the forced swimming test (FST) (Figure 1; Fisher’s LSD post hoc: *p* < 0.05). Moreover, an increase in the swimming time by about 30% versus the ESC (10 mg/kg) group was also observed (Fisher’s LSD post hoc: *p* < 0.05).

We have also tested the involvement of the GALR2 in the GAL(1-15)-ESC interaction with the GALR2 antagonist M871 in the FST. M871 (3 nmol) significantly blocked the GAL(1–15)-induced reduction in the immobility time (Fisher’s LSD post hoc: *p* < 0.001) (Figure 1A), and the GAL(1–15)-induced increase in the swimming time (Fisher’s LSD post hoc: *p* < 0.05) (Figure 1B) found after coadministration of GAL(1-15) and ESC (10 mg/kg) in the FST.

**Figure 2:**
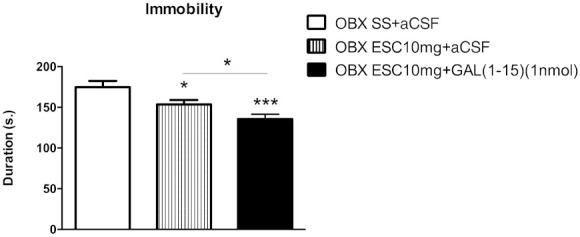
Behavioural effects of the coadministration of effective dose of ESC (10 mg/kg) and GAL(1-15) (1 nmol) in the tail suspension test (TST). Saline or ESC was administered intraperitoneal (ip) 23, 5, and 1 h before the test. aCSF or GAL(1-15) was injected intracerebroventricular (icv) 15 min before the test. Data represent mean ± SEM of immobility time in TST during the 6 min test period (n = 8–10 rats per group). * *p* < 0.05 versus OBX SS+aCSF, *** *p* < 0.001 versus OBX SS+aCSF group; according to one way ANOVA followed by Fisher multiple comparison test.

In the tail suspension test (TST), the three ip injections of ESC (10 mg/kg) significantly reduced the immobility behaviour recorded during the 6 min of the test (one-way ANOVA, F_2,24_ = 9.12, *p* = 0.001, Fisher’s LSD post hoc: *p* < 0.05) (Figure 2). Moreover, the coadministration of ESC (10 mg/kg) and GAL(1-15) (1 nmol) induced antidepressant-like effects with a significant decrease in the immobility time compared with ESC (Fisher’s LSD post hoc: *p* < 0.05) (Figure 2).

No statistically significant differences were observed in the FST and TST between OBX and SHAM rats (Supplementary Table S3).

#### 2.1.2. siRNA 5HT1AR Knockdown in OBX Rats Validates the Involvement of 5-HT1AR in the Effects Induced by GAL(1-15)

**Figure 3:**
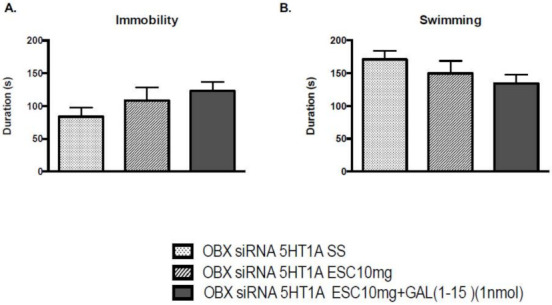
Behavioural effects of knockdown 5HT1A model of the coadministration of effective dose of ESC (10 mg/kg) and GAL(1-15) (1 nmol) in the forced swimming test (FST). Saline or ESC was administered intraperitoneal (ip) 23, 5, and 1 h before the test. aCSF or GAL(1-15) was injected intracerebroventricular (icv) 15 min before the test. Data represent mean ± SEM of immobility (**A**) and swimming (**B**) time in FST during the 5 min test period (n = 8–13 rats per group). There are no significant differences according to one way ANOVA followed by Fisher multiple comparison test.

In OBX rats, the downregulation of 5-HT1AR by siRNA did not affect any parameter in the FST (Supplementary Table S4). However, the decrease in 5-HT1AR was sufficient to block GAL(1-15) enhancement of the antidepressant-like effects mediated by ESC (Figure 3). Thus, the coadministration of GAL(1-15)+ESC lacked effect on the immobility (one-way ANOVA, F_2,27_ = 1.60, *p* = 0.218) and swimming time (one-way ANOVA, F_2,27_ = 1.49, *p* = 0.243) in the FST (Figure 3).

GAL(1-15) (1 nmol) injected alone lacked effects in the FST in OBX knockdown 5HT1AR animals compared to control groups (OBX-Delivery Media; OBX siRNA 5-HT1AR) in terms of immobility and swimming time (Supplementary Table S4).

### 2.2. c-Fos Immunohistochemistry

**Figure 4:**
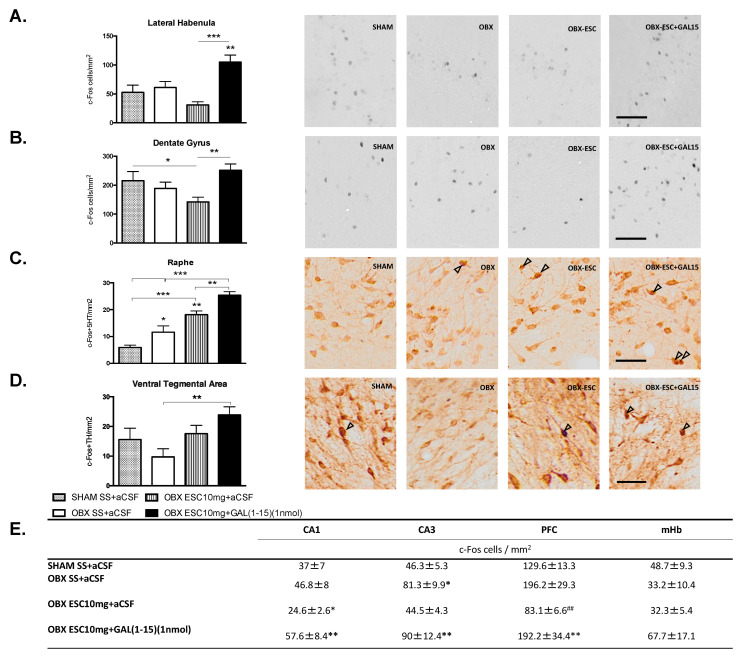
Effects of ESC10 mg and GAL(1-15) (1 nmol) alone or in combination on c-Fos expression. Data represent mean ± SEM of c-Fos cells/mm^2^ in dorsal hippocampus (dentate gyrus (DG), CA1, CA3), prefrontal cortex (PFC), lateral habenula (LHb), and medial habenula (mHb). The mean ± SEM of c-Fos/5HT in the dorsal raphe (DR) and c-Fos/TH cells/mm^2^ in the ventral tegmental area (VTA) is also shown. One way ANOVA followed by Fisher multiple comparison test (n = 3–5 in each group). In subfigures (**A**–**D**), a representative photomicrograph illustrating the different treatments are also shown. Scale bar = 100 μm. In (**A**) ** *p* < 0.01 versus OBX SS+aCSF and SHAM SS+aCSF and *** *p* < 0.001 versus OBX ESC10 mg/kg+aCSF. In (**B**) * *p* < 0.05 SHAM SS+aCSF versus OBX ESC10 mg/kg+aCSF and ** *p* < 0.01 versus OBX ESC10 mmg/kg+aCSF; In (**C**) * *p* < 0.05 versus SHAM SS+aCSF and ** *p* < 0.01 versus OBX SS+aCSF; In (**D**) ** *p* < 0.01 versus OBX SS+aCSF; In (**E**): In CA1, * *p* < 0.05 versus OBX SS+aCSF and ** *p* < 0.01 versus OBX ESC10 mg/kg+aCSF. In CA3, * *p* < 0.05 versus OBX ESC10 mg/kg+aCSF and SHAM SS+aCSF, and ** *p* < 0.01 versus OBX ESC10 mg/kg+aCSF and SHAM SS+aCSF. In PFC ** *p* < 0.01 versus OBX ESC10 mg/kg+aCSF and ## *p* < 0.01 versus OBX SS+aCSF.

We have analysed c-Fos immunoreactivity (c-Fos-IR) 90 min after the coadministration of icv GAL(1-15) and the three ip injections of ESC (10 mg/kg) in OBX rats in several nuclei involved in MDD: Dorsal Hippocampus, PFC, LHb and mHb (Figure 4A,B,E).

Double immunohistochemical staining of 5-HT and c-Fos-IR or TH and c-Fos-IR were used to study the specific cell activation in the DR and VTA, respectively, after GAL(1-15)+ESC (Figure 4C,D).

As seen in Figure 4 the coadministration of GAL(1-15)+ESC in OBX rats produce a significant increase in the number of c-Fos-IR profiles compared with ESC group in the DG (one-way ANOVA, F_3,15_ = 4.38, *p* = 0.020, Fisher’s LSD post hoc: *p* < 0.01), CA1 (one-way ANOVA, F_3,13_ = 4.54, *p* = 0.021, Fisher’s LSD post hoc: *p* < 0.01), CA3 (one-way ANOVA, F_3,14_ = 6.29, *p* = 0.006, Fisher’s LSD post hoc: *p* < 0.01), PFC (one-way ANOVA, F_3,14_ = 4.59, *p* = 0.019, Fisher’s LSD post hoc: *p* < 0.01), and LHb (one-way ANOVA, F_3,15_ = 9.29, *p* = 0.001, Fisher’s LSD post hoc: *p* < 0.001).

Similarly, the number of c-Fos-IR in serotoninergic cell bodies in the DR after icv GAL(1-15)+ESC in OBX rats was significantly increased in comparison with c-Fos-IR in serotoninergic cell bodies in the ESC group (one-way ANOVA, F_3,16_ = 28.67, *p* = 0.0001, Fisher’s LSD post hoc: *p* < 0.01) (Figure 4C). Moreover, in VTA, the number of c-Fos-IR in TH cell bodies after icv GAL(1-15)+ESC in OBX rats was significantly increased in comparison with c-Fos-IR in TH cell bodies in the OBX saline group (one-way ANOVA, F_3,16_ = 3.64, *p* = 0.035, Fisher’s LSD post hoc: *p* < 0.01) (Figure 4D).

ESC (10 mg/Kg) in OBX rats induced a significant decrease in the number of c-Fos-IR profiles in the CA1 (Fisher’s LSD post hoc: *p* < 0.05) and PFC (Fisher’s LSD post hoc: *p* < 0.01) compared with OBX saline group (Figure 4E) and in the DG (Fisher’s LSD post hoc: *p* < 0.05) compared to the Sham group (Figure 4B). On the contrary, the number of c-Fos-IR in serotoninergic cell bodies in the DR after ESC in OBX rats was significantly increased in comparison with c-Fos-IR in serotoninergic cell bodies in the OBX saline group (Fisher’s LSD post hoc: *p* < 0.01) (Figure 4C).

**Figure 5:**
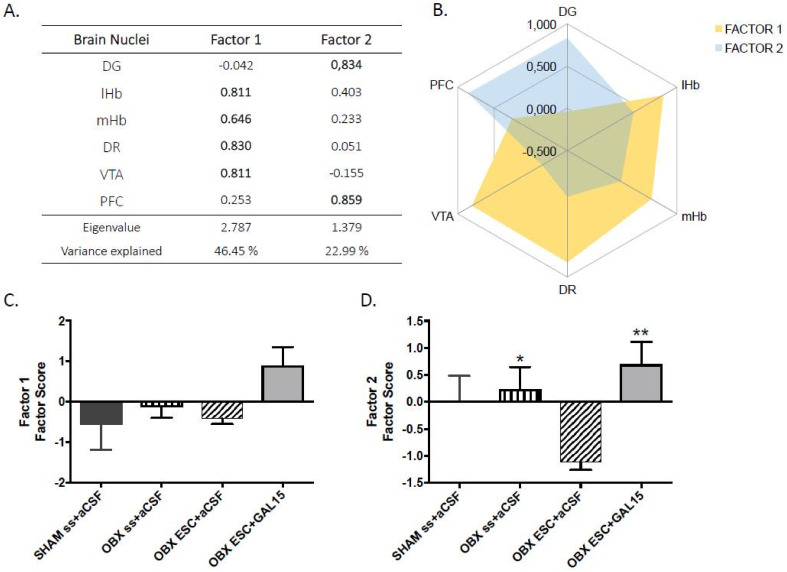
General regions extracted in principal component analyses. (**A**) The PCA of the number of c-Fos positive nuclei in a brain nucleus of revealed two independent dimensions (factors): Factor 1 and Factor 2. (**B**) Factor 1 was obtained from measures registered in habenula lateral (LHb), habenula medial (mHb), ventral tegmental area (VTA), and dorsal raphe (DR). Factor 2 was obtained from measures registered in dentate gyrus (DG) and prefrontal cortes (PFC). A brain nucleus was considered to be included in a factor when its loading was > 0.5 in absolute value (highlighted in bold). (**C**,**D**) Data represent the mean of the factor scores of the subjects in each one of the networks ± SEM of Factor 1 and 2 in SHAM SS+aCSF, OBX SS+aCSF, OBX ESC10 mg+aCSF, and OBX ESC10 mg+GAL(1-15)(1 nmol) (n = 4–5 rats per group). In Factor 1 there are no significant differences according to one way ANOVA followed by Fisher multiple comparison test. In Factor 2 * *p* < 0.05 versus OBX SS+aCSF and ** *p* < 0.01 versus OBX ESC10mg+aCSF according to one way ANOVA followed by Fisher multiple comparison test.

The PCA revealed two independent factors representing the functional brain modules or networks underlying c-Fos-IR that explained ~70% of the total variance (Figure 5A,B). The first factor encompassed LHb, mHB, DR, and VTA (46.45% of explained variance) while the second factor was composed of DG and PFC (22.99% of explained variance). PCA statistical assumptions were satisfied allowing its use and interpretation (Kaiser–Meyer–Olkin, KMO = 0.591; Bartlett’s sphericity test: X2(15) = 31.938, *p* = 0.007). To determine the relevance of each brain network (factor) in each of the experimental groups (SHAM, OBX saline, OBX-ESC, OBX-ESC+GAL(1-15)), we calculated the factor scores of the subjects in each one of the networks and a one-way ANOVA was performed in each of the factors. Regarding to the first brain network (Factor 1), we observed that the OBX-ESC+GAL(1-15) group is different from the rest of the groups, although it did not reach the statistical significance (Figure 5C). In the case of the second brain network (Factor 2), there were significant group differences (F_3,13_ = 3.97, *p* < 0.032). The post hoc test shows that the OBX-ESC+GAL(1-15) group presents a higher activity (c-Fos-IR) compared with the ESC group (*p* < 0.005) (Figure 5D).

## 3. Discussion

The current study results described that in OBX rats, GAL(1-15) enhances the antidepressant-like effects induced by ESC in behavioural despair tests; GALR2 was involved in these effects since the GALR2 antagonist M871 blocked GAL(1-15) mediated actions in the FST. Significantly 5HT1AR participates in the GAL(1-15)/ESC interactions; the downregulation of 5-HT1AR by siRNA was sufficient to block GAL(1-15) enhancement of the antidepressant-like effects mediated by ESC. Our immunohistochemistry and PCA analysis suggest that two functional networks are involved in these effects; one of them includes the LHb, mHB, DR, and VTA, and the other consists of the DG and PFC inducing the OBX-ESC+GAL(1-15) group a higher number of c-Fos-IR profiles in both networks.

In the present study, OBX rats exhibited the characteristic hyperactivity in the open field paradigm and increased BDNF and TRKb mRNA levels as previously described [26,27,29,30]. Moreover, the ablation of the olfactory bulbs lacked an effect on the mRNA levels of GALR1 or GALR2 in the PFC, as we have previously described in the hippocampus [26].

We have described for the first time a significant decrease in the Homer1A mRNA levels in the PFC. The Homer1A is a neuronal immediate-early gene involved in regulating synaptic plasticity and suggests being involved in depression-like behaviour (for revision, see [31]). Mice subjected to the model of chronic depression show reduced Homer1A expression in the PFC [31] and specific siRNA knockdown of Homer1A in PFC enhances depressive-like behaviour [31], suggesting that Homer1A expression specifically in the PFC is inversely correlated to the depressive-like behaviour [31]. The decrease observed in the depressive OBX model in the Homer1A mRNA levels in the PFC confirmed the relation of this immediate-early gene with depression behaviour.

Among behavioural tests, we have selected two tests related to despair: FST and TST. Behavioural tasks that assess despair commonly measure an animal´s survival or lack thereof. FST is a reliable tool that has been used globally not only in industrial settings for screening and discovering new antidepressant substances but also in complementary depression medicine research, and some over the FST prefer the TST due to the reduced chance of inducing hypothermia and the reduced variability that could be accounted for by differences in water temperature or equipment used across labs [32].

Our results indicate that in OBX rats, GAL(1-15) enhanced ESC-effects in both behavioural tests, the FST and the TST. These results are in line with our previous work; GAL(1-15) improved the anti-depressive effects induced by the SSRIs fluoxetine (FLX) not only in naïve animals [24,25] but also in OBX animals [26]. ESC is an SSRI with more efficacious than FLX in reducing the depressive symptoms for the acute phase treatment of major depression [28] and widely study in augmentation therapy in treatment-resistant depression (TRD) with aripiprazole [33,34] or buspirone [35]. Our results confirm a potent effect of the combination GAL(1-15) with SSRIs in reversed depressive symptoms and open up the possibility to use this combination as augmentation therapy in MDD.

In our study, ESC induced a reduction in immobility in the TST in OBX animals. This result agrees with previous ESC acute studies in rodents using several ESC doses [36]. However, in this work, ESC lacked an effect in FST. Although this is the first study describing the impact of ESC in OBX animals in the FST, in naïve rats, ESC at 10 mg shows the variability of response: no effect or a decrease in the FST [37,38]; This variability has been explained by the different pattern of ESC used. Since in this work we have used a subchronic injection pattern, we will need to address the effect of GAL(1-15) in chronically administered ESC in OBX rats in future experiments.

In our OBX model, we did not find any effect in the FST or in the TST in OBX-saline versus sham rats. These results suggest that in our study, the hyperactivity observed in the open field test (OFT) is not affecting the FST or the TST behaviour test. The lack of effect in the FST is consistent with other studies in OBX animals [39] although other authors have found an increased in the immobility in this test [27]. This variability observed in this test indicates that the FST is not a representative behavioural domain in the OBX model [32].

Our results obtained in the siRNA 5HT1AR knockdown in OBX rats confirm the critical role of 5-HT1AR in the effects induced by GAL(1-15). GAL(1-15) 1 nmol alone has no effect in the FST in siRNA 5HT1AR knockdown OBX model. This result is in agreement with the lack of effect of GAL(1-15) 1 nmol in the FST in the immobility or swimming in OBX rats (Data not shown). The decrease in 5-HT1AR was sufficient to block GAL(1-15) enhancement of the antidepressant-like effects mediated by ESC. These results agree with our previous work in naïve animals [24,25] and OBX animals [26]. We have described that the GAL(1-15)/FLX interactions in the OBX animals involves the 5-HT1AR in the hippocampus at the plasma membrane and transcriptional levels [26]. All these data reinforce our previous hypothesis, the existence of a trimeric GALR1-GALR2-5-HT1AR heteroreceptor complex [17,23,24,25] that could be a pivotal point to understand the effects of GAL(1-15)-SSRI interaction in the OBX animal depression model. In such complex altered allosteric receptor–receptor interactions can develop with the ability of the GALR1-GALR2 component to enhance the 5-HT1AR protomer signalling [25].

Because not only 5-HT1AR, but also 5-HT4, 5-HT2A, 5-HT3, and 5-HT7 receptors are involved in modulating the effects of antidepressant treatments, the participation of other 5-HT receptor subtypes in the interaction cannot be excluded.

About the areas involved in the GAL(1-15)-SSRI interaction in the OBX animal, the coadministration of GAL(1-15)+ESC in OBX rats produce a significant increase in the number of c-Fos-IR profiles in several nuclei involved in MDD: dorsal hippocampus, PFC, and LHb. Moreover, an increase in the number of c-Fos-IR serotoninergic cell bodies in the DR and the c-Fos-IR TH cell bodies in the VTA was observed after GAL(1-15)+ESC.

PCA described two functional brain networks or factors in this study, although we must be cautious because our research is not extensive. In relation with the functional network comprised of DG and PFC (Factor 2), we have observed in two previous independent works that both nuclei were involved in the GAL(1-15)–FLX interaction in naïve rats [24,25], and also the DG was a crucial area in the GAL(1-15)–FLX interaction in OBX rats [26]. The fact that PCA associate these two nuclei in one network open up the possibility that the participation of both areas is interrelated.

With regard to the other functional network (Factor 1), comprised for LHb, DR, and VTA, we have previously described an essential role of DR in the depression and anxiety-like behaviours induced by GAL(1-15) [22], and also this nucleus participates in the enhancement of the antidepressant-like actions of a 5-HT1AR agonist in the FST by GAL(1-15) [23]. The VTA has also been described to be involved in GAL(1-15)-mediated effect; in fact, we have proposed that the VTA through the VTA-limbic-cortical DA system is responsible for the anhedonia-like behaviour induced by GAL(1-15) [21]. Our results in this study confirmed the important role of these two nuclei in GAL(1-15)-mediated action.

It is of high interest the participation of the LHb in the first functional brain network. LHb has been recently associated with depressive symptoms, such as helplessness and anhedonia [40,41]. In humans, functional studies revealed its hyperactivity in individuals with major depression [42], and deep brain stimulation to the inactive LHb reported either full revision [43] or alleviation of MDD [44]. Additionally, in animal models, Ketamine, the new rapid antidepressant drug, acts by suppressing the activity of the LHb neurons [45]. Circuitry-wise, the LHb is one of the few brain regions that control both the dopaminergic system and the serotonin system. LHb acts a relay station that interconnects the limbic forebrain with depression-related monoaminergic centers, including the VTA and the DR [40,41]. The circuit configuration downstream of the LHb suggests that during depression, hyperactivation of the LHb may suppress DA and 5HT neurons through the GABAergic RMTg neurons and local interneurons [40,41]. In addition, one major input comes as a reciprocal feedback from the monoaminergic centers, including the VTA and the DR. The LHb-targeting VTA neurons include a TH-positive population, and it is clear that this population release GABA and suppress LHb output [46]. Moreover, serotonin projections form the DR to the LHb suppress its excitability [47] and optogenetic activation or inhibition of the DR-LHb pathway alleviates or induces depressive-like behaviours, respectively [47].

Our results showing that GAL(1-15) enhance the antidepressant-like effects induced by ESC could be explained by activating the DR-LHb and VTA-LHb pathways. However, a detailed analysis of these circuits should be analysed in future studies.

In conclusion, our results indicate a potent effect of the combination GAL(1-15) with SSRIs in reversed depressive symptoms in the animal model of chronic depression. The results open up the possibility to use GAL(1-15) in combination with SSRIs as a novel strategy for treatment of depression.

## 4. Materials and Methods

### 4.1. Animals

Male Sprague Dawley rats were obtained from CRIFFA (bodyweight 225–250 g), maintained in a 12 h dark/light cycle under control the conditions of humidity (55–60%) and temperature (22 ± 2 °C). The animals were given ad libitum access to food and water. All animal experimentation was conducted following the University of Málaga Guidelines for the Care and Use of Laboratory Animals (Ethic Code: 22/05/2017/066).

**Figure 6:**
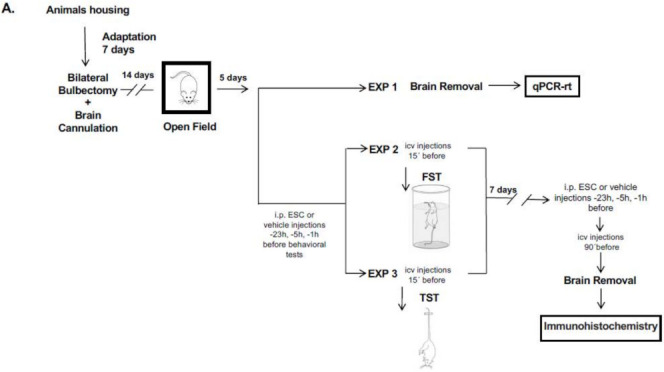

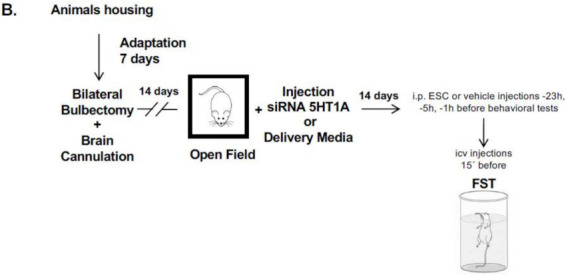
Diagram of the complete experimental schedule. (**A**) After, bilateral olfactory bulbectomy, all animals had a recovery period of 14 days and then we determine their hyperactivity using the open field test (OFT). One set of animals were randomly selected and their brains were collected to perform the experiments of qPCR-rt. In other set of animals, we evaluated the effects of different pharmacological treatments in the forced swimming test (FST) or tail suspension test (TST). After the behavioural tests we collected the brain of the animals to perform the experiments of immunohistochemistry. (**B**) after bilateral olfactory bulbectomy, all animals had a recovery period of 14 days and then we determine their hyperactivity using the open field test. After we injected siRNA 5HT1A or delivery media and all animals had a recovery period of 14 days and then we evaluated the effects of different pharmacological treatments in the FST.

After the accommodation period, animals underwent either olfactory bulbectomy (OBX) or sham surgery. The OBX procedure and the stereotaxically implanted guide cannula has been described previously [26] (Supplementary Materials). On day 14 post-surgery, we confirmed the validity for the lesions by assessing increased activity in the open field. A pictogram of the entire protocol is represented in Figure 6.

Five days after the open field, animals were randomly divided into three experiments. In the first experiment, we analysed the relative mRNA expression of GALR1, GALR2, 5-HT1AR, BDNF, TRKb, Homer1A, and RAB5 in a set of OBX and Sham-operated rats.

In the other independent experiments, rats were assessed in the forced swimming test (FST) or the tail suspension test (TST). We have evaluated the effects of the administration of ESC(10 mg/Kg) and GAL(1-15)(1 nmol) [24,25], alone or in combination in both tests. The ESC or vehicle was injected three times intraperitoneally (ip) at the doses of 10 mg/kg 23, 5, and 1 h before the beginning of the tests; this pattern of injection was shown to produce effects of ESC in the FST similar to those obtained after subchronic treatment [37]. The sample size and the dose of GAL(1-15)(1 nmol) were estimated based on our previous works where both behavioural tasks (FST and TST) in Sprague–Dawley rats were used [22].

In the FST, we also determined the involvement of GALR2 in the effect of GAL(1-15) on ESC-mediated action in rats that received three injections of ip ESC(10 mg/kg) and a single icv injection of GAL(1-15)(1 nmol) and the GALR2 antagonist M871(3 nmol) in combination. OBX animals were randomly divided into groups to assess the treatments.

After the behavioural experiments, rats were euthanised to analyse the c-Fos expression 90 min after the administration of ESC(10 mg/Kg) and GAL(1-15)(1 nmol), alone or in combination in the dorsal hippocampus, PFC, lateral (LHb) and medial (mHb) habenula. Moreover, double immunohistochemical staining of 5-hydroxytryptamine (5-HT) and c-Fos-IR or tyrosine hydroxylase (TH) and c-Fos-IR were used to study the specific cell activation in the DR and the VTA.

To analyse the roles of 5-HT1AR, in a second set of experiments we have generated siRNA 5HT1A knockdown rats as previously described [22,25,48].

Using real-time quantitative PCR, we have performed a time course of 5-HT1A mRNA in the dorsal hippocampus, and we had also conducted a time course of 5HT1A protein expression using quantification of immunohistochemical staining for 5HT1A in the DR. The time course curve indicated a maximal reduction in 5HT1A receptor protein expression 14 days after the siRNA 5HT1A injection (Figure S1).

Briefly, after the accommodation period, animals underwent either olfactory bulbectomy (OBX) or sham surgery and were stereotaxically implanted with a guide cannula. On day 14 post-surgery, we confirmed the validity for the lesions by assessing increased activity in the open field. The day after the open field test, the rats received an icv injection of 5 μg (0.35 nmol) of Accell Smart pool siRNA 5HT1A (Figure 6B). Fourteen days later (the time required to reduce the levels of the 5HT1A receptors), we have evaluated the effects of the administration of ESC(10 mg/Kg) and GAL(1-15)(1 nmol) alone or in combination with the FST. A pictogram of this protocol is represented in Figure 6B.

Detailed descriptions of animals, surgical procedures, and administration of substances, drugs, and knockdown model are available in the Supplementary Materials.

### 4.2. Behavioral Assessment

Open field test: Rats were individually placed in the center of the arena and allowed to explore freely. Their activity was videorecorded over a 5 min period and the behaviour was analysed off-line using the video tracking software EthovisionXT (see Supplementary Materials for details).

Forced swimming test: Two swimming sessions were conducted: a 15 min pretest followed 24 h later by a 5 min test. The total duration of immobility behaviour and swimming and climbing were recorded during the second 5 min. The administration of drugs was performed between sessions (see Supplementary Materials for details).

Tail suspension test: Rats were individually hung upside down placed in the center. The total duration of immobility behaviour was recorded during 6 min. The administration of drugs was 23, 5, and 1 h before the test (see Supplementary Materials for details).

### 4.3. Immunohistochemistry and Inmunofluorescence

Then, 90 min after the drugs administration, rats were anaesthetised with sodium pentobarbital (Mebumal; 100 mg/kg body weight, i.p.) and intracardially perfused with 200 mL isotonic ice-cold saline phosphate buffer followed by 200 mL of fixation fluid 4% paraformaldehyde (*w/v*) in saline 0.1 M sodium PB ((PBS), pH 7.4). The brains were removed, postfixed for 12 h in the same fixative and cryoprotected in sucrose (30% at 4 °C). Brainstem 30 μm coronal sections were obtained on a cryostat. The sections were sequentially incubated with primary antibodies (1/1800 c-Fos mouse polyclonal antibody, sc-271243, Santa Cruz Biotech, Dallas, Texas, USA; 1/20,000 5-HT rabbit monoclonal antibody, 20080, INCSTAR, Stillwater, MN, USA; 1/2500 TH mouse monoclonal antibody, T1299, Sigma and in inmunofluorescence were incubated with 1/500 5-HT1A mouse monoclonal antibody, MAB11041, Merck, Germany) (see Supplementary Materials for further details).

The immunoreactivity was analysed in DG, CA1, and CA3 of the hippocampus, PFC, LHb, mHb. Double immunohistochemical staining of 5-HT and c-Fos-IR or TH and c-Fos-IR were used to study the specific cell activation in the DR and VTA.

### 4.4. Genes Expression by rt-PCR

Rats were euthanised by decapitation 1 h after drugs administration, and the brains were rapidly removed and frozen until use. The nuclei dissections were conducted as described [21]. The procedure to perform RNA isolation and RT-PCRs was described previously [21,22,25]. The primer sequences used to evaluate the mRNA expression levels of the genes GADPH, 5HT1A, Rab5, BNDNF and TRKb in the dorsal hippocampus, and 5-HT1A, Homer1A, GALR1, and GALR2 in the prefrontal cortex are shown in the Supplementary Materials.

### 4.5. Statistical Analysis

Data are presented as the mean ± standard error of the mean, and sample numbers (n) are indicated in figure legends. All data were analysed using GraphPad PRISM 8.0 (GraphPad Software, San Diego, CA, USA). For comparing two experimental conditions, Student´s unpaired t-tests were performed. For comparing more than two groups, one-way analysis of variance (ANOVA) was performed. Fisher’s least significant difference (LSD) comparison post-test was performed only when the F ratio in the one-ANOVA was statistically significant. Differences were considered statistically significant at *p* < 0.05 (* *p* < 0.05, ** *p* < 0.01, *** *p* < 0.001).

A principal components factorial analysis (PCA) with varimax rotation was also performed to extract the independent dimensions (i.e., factors) from the c-Fos-IR data. Eigenvalue > 1 was chosen as criterion for component extraction and a factor score (i.e., a standardised value indicating the relative position of each animal in each factor) was computed by the regression method (SPPS Statistics 20, (IBM Corporation, Armonk, NY, USA). Only measures with a saturation greater than 0.5 in absolute value were included in a factor.

## Data Availability

The data presented in this study will be openly available in RIUMA-University of Malaga once the manuscript is accepted for publication.

## Supplementary Materials

Supplementary materials are available online at https://www.mdpi.com/article/10.3390/ijms221910848/s1.
