# Supplementary material for: Galanin(1-15) Potentiates the Antidepressant-like Effects Induced by Escitalopram in a Rat Model of Depression
Source: Int J Mol Sci. 2021 Oct 7;22(19):10848. doi: 10.3390/ijms221910848 (PMC8509384; doi:10.3390/ijms221910848)
Supplement: Supplementary file 1 [file ijms-22-10848-s001.zip › ijms-1377764-supplementary.pdf]

Supplemental Methods

Animals

Male Sprague Dawley rats (bodyweight 225–250g, age eight weeks) were obtained from Criffa and maintained in a humidity- and temperature-controlled (20–22 ºC) room. The rats were kept on 12-/hour light/dark process and had free access to food pellets and tap water. Experimental procedures were approved by the Institutional Animal Ethics Committee of the University of Málaga, following the European Directive (86/609/EEC) and Spanish Directive (Real Decretory 53/2013).

Surgical Procedures.

Briefly, rats were anaesthetized with isoflurane (ISOFLO® ZOETIS ABOTT Liquid gaseous anaesthetic, 100% Isoflurane) 5% in induction chamber and maintenance of 1.5-2.5% Isoflurane mask, and placed in the stereotactic instrument. Following exposure of the skull, we drilled 2mm diameter holes at the points 7mm anterior to bregma and 2mm on either side of the mid-line. Then olfactory bulbs were removed by suction, and holes were filled with a haemostatic sponge to stop the bleeding (Flores-Burgess 2021). Sham-operated animals were treated in the same way, but the bulbs were left intact.

After bulbectomy or sham-bulbectomy, a chronic 22-gauge stainless-steel guide cannula was implanted in all animals into the right lateral cerebral ventricle (+1.4mm lateral, -1mm posterior to bregma, and 3.6mm below the surface of the skull) (Paxinos, 1986). This protocol has been described previously (Diaz-Cabiale et al., 2011). Animals were individually housed after surgery and had a recovery period of 14 days before the Open Field Test (OFT). We confirmed the validity of the lesions by assessing increased activity in this test.

Administration of substances and drugs.

Intracerebroventricular (icv) administration of substances was performed for 1min in a volume of 5μl using a 26-gauge stainless-steel injection cannula connected to a Hamilton syringe via PE-10 tubing with special care that during the process the dilutions were

administered fluently and without overflowing. All rats that received the icv injections were assumed to have proper cannula placement, and none were excluded. For behavioural experiments, ESC or vehicle was injected three times intraperitoneally (ip) at the doses of 10 mg/kg 23, 5 and 1h before the beginning of the tests; this pattern of injection was shown to produce effects of ESC in the Forced Swimming Test (FST) similar to those obtained after subchronic treatment (Reed et al., 2008). Icv GAL(1-15) 1nmol alone or combined with the GALR2 antagonist M871 3nmol was injected 15min before the beginning of the tests.

We used the following drugs: Escitalopram Oxalate (Sigma-Aldrich, San Luis, EEUU) and GAL(1-15) and the GALR2 antagonist M871 (TOCRIS, Bristol, Reino Unido).The solutions were prepared using artificial cerebrospinal fluid (aCSF). For ip administration of ESC, solutions were dissolved in 0.9% saline solution and were administered in a volume of 0.1 ml/100 g body weight.

Behavioral assessment

Open Field Test. On the 14th day after the surgery, rats were individually placed in the center of the arena (100x100x50cm) and allowed to explore freely; their behaviour was recorded over a 5min period by a ceiling-mounted video camera. Their activity (total distance) was analyzed using the video tracking software EthovisionXT (Noldus Information Technology Inc, Leesburg, VA). After each trial, all surfaces were cleaned with a paper towel and 70% ethanol solution (Millón et al., 2015).

Forced Swimming Test. Animals were individually placed in a vertical glass cylinder (50cm height, 20cm diameter) containing water (25ºC) to a height of 30cm. Two swimming sessions were conducted: a 15min pretest followed 24h later by a 5min test. The total duration of immobility behavior and swimming were recorded during the second 5min. Immobility was defined as floating passively in an upright position in the water with only slight movements necessary to keep the head above the water surface.. The administration

of drugs was performed between sessions. All tests were performed between 13:00 and 16:00h during the light cycle (Millón et al., 2015; Flores-Burgess et al., 2017).

Tail Suspension Test. The rat was hung upside down using an adhesive tape to fix its tail to a rope through an eyebolt at 60 cm. The animal was considered immobile when it was not making any movements or struggling, attempting to catch the adhesive tape, body torsions, or jerks (Hinojosa et al., 2006; Yan et al., 2014 and Millón et al,. 2015).

siRNA 5HT1A knockdown rats

Knockdown rats were generated as previously described (Millon et al., 2015). Using real-time quantitative PCR, we have previously performed a time course of 5HT1A mRNA in the Hippocampus. We had also conducted a time course of 5HT1a protein expression using quantification of immunofluorescence staining for 5HT1A in Dorsal Raphe (DR).

c-Fos immunohistochemistry

The sections were processed free-floating. Endogenous peroxidase activity was removed by incubating the sections with 10% H2O2 and 10% methanol for 30 min. After blocking and permeabilization with 0,2% Bovine Serum Albumin (Sigma, Spain) with 0.5% Triton X-100 (10 min, room temperature), the sections were incubated with a mouse polyclonal antibody (Santa Cruz Biotech. sc-271243, USA) raised against the c-Fos protein (1/800). Overnight incubation at 4 °C was performed in 0.01 M PBS containing 0.5% Triton X-100. The sections were washed 3 times in PBS and incubated with biotinylated anti-mouse polyclonal goat (Dako E 0433) (1/600) for 1 h at room temperature. The immunostaining was performed with streptavidin peroxidase (SIGMA E2886) (1/1000) for 1 h at room temperature. The chromogen used was 0.05% 3-30-diaminobenzidine tetrahydrochloride (DAB) (Sigma, Spain) intensified with nickel chloride hexahydrate (Sigma, Spain) 0.04% (w/v), giving darker black nuclei staining.

Sections from every animal from each experimental group were processed simultaneously. Omission of the primary antibody resulted in no detectable staining. After mounting the sections on gelatin-chromium coated slides, the sections were dehydrated and coverslipped with DPX (Panreac, Barcelona, Spain). Thus, every section was numbered according to the rostrocaudal level (Paxinos, 1986).

Images are taken with an Olympus VS120 microscope with a PLAN AP 10x objective. The areas of interest are manually cut out, and a Gaussian filter is applied to them. A Find_Maxima function that locates the c-Fos marks is used to the resulting image with the FIJI program (Schindelin et al., 2012).

c-Fos/5HT immunohistochemistry

Immunostaining of c-Fos was performed under the same conditions as the c-Fos previously described.

5-HT immunostaining was performed under the same conditions as c-Fos but using 0.9% saline Tris buffer 0.1 M (pH 7.6) instead of saline phosphate buffer. The primary antibody was a rabbit monoclonal antibody raised against 5-HT (20080, INCSTAR, USA) (1/20000). Nickel chloride was not added to the chromogen solution in the second incubation for immunostaining in order to get a brownish reaction. Sections from every animal from each experimental group were processed simultaneously. Omission of the primary antibody resulted in no detectable staining. After mounting the sections on gelatin-chromalum coated slides, the sections were dehydrated and coverslipped with DPX (Panreac, Barcelona, Spain). Thus, every section was numbered according to the rostrocaudal level (Paxinos, 1986).

Images are taken with Olympus VS120 microscope with a PLAN AP 10x objective. The areas of interest are manually cut out and manually counted c-Fos and 5HT marks with the cell counter, tool of the FIJI program, (Schindelin et al., 2012).

c-Fos/TH immunohistochemistry

Immunostaining of c-Fos was performed under the same conditions as the cFos previously described.

TH immunostaining was performed under the same conditions as c-Fos. The primary antibody was a mouse monoclonal antibody raised against TH (T1299, Sigma) (1/2500). Nickel chloride was not added to the chromogen solution in the second incubation for immunostaining to get a brownish reaction. Sections from every animal from each experimental group were processed simultaneously. Omission of the primary antibody resulted in no detectable staining. After mounting the sections on gelatin-chromalum coated slides, the sections were dehydrated and coverslipped with DPX (Panreac, Barcelona, Spain). Thus, every section was numbered according to the rostrocaudal level (Paxinos, 1986).

Images are taken with Olympus VS120 microscope with a PLAN AP 10x objective. The areas of interest are manually cut out and manually counted c-Fos and 5HT marks with the cell counter, tool of the FIJI program, (Schindelin et al., 2012).

Immunofluorescence

The procedures have been used previously (Millon et al., 2015). Primary antibodies directed to anti-serotonin receptor 1A monoclonal antibody (clone 19A9.2 mouse MAB11041, Merck, Germany, 1/500) were used.

Genes expression by rt-PCR

Total RNA was isolated from the hippocampus using RNeasy Lipid Tissue Kit (Qiagen), and cDNA was obtained using a Reverse Transcriptase Core kit (Eurogentec). These two steps were performed according to the manufacturer's instructions. All the analysis was conducted in triplicate using LightCycler® Multiplex Masters in a PCR Light Cycler 96 (Roche®), and relative gene expression was determined using the double delta Ct method and normalized to measures of glyceraldehyde-3-phosphate dehydrogenase (GAPDH) mRNA. Primers used in this study was purchased predesigned from the Sygma-Aldrich® and was previously used. GAPDH was used as housekeeping. The primer sequences used in this study are:

GAPDH-Forward: 5´-GCTCTCTGCTCCTCCCTGTTC;

GAPDH-Reverse: 5´-GAGGCTGGCACTGCACAA;

GAL2R-Forward: 5´-AACAGGAATCCACAGACC;

GAL2R-Reverse: 5´-CCCTTTGGTCCTTTAACAAG;

GAL1R-Forward: 5´-AAAACTGGACAAAACTTAGCC;

GAL1R-Reverse: 5´-GGATACCTTTGTCTTTGCTC.

5-HT1AR-Forward: 5´-AACTATCTCATCGGCTCC;

5-HT1AR-Reverse: 5´- ACATCCAGGGCGATAAAC;

BDNF-Forward: 5´-GGAGACGAGATTTTAAGAC;

BDNF-Reverse: 5´-CCATAGTAAGGAAAAGGAT

HOMER 1A-Forward: 5´-CTGCTCCAAAGGAAAGCCTTGC;

HOMER 1A-Reverse: 5´-AAACAACCTTCAATGCTGACGG;

TRKB-Forward: 5´-TCTGTACCAAAATACACGTC;

TRKB-Reverse: 5´-TTTGGGTTTGTCTCATAGTC;

RAB5-Fordward: 5′-AAAAGAGCTGTTGACTTCC;

RAB5-Reverse: 5′-AGGTCTACTCCTCTTCCTC.

Table S1. Effects between OBX and SHAM rats in the OFT.

	**Distance (cm)**	**Velocity (cm/s)**
**SHAM**	3422±154,1	11,4±0,5
**OBX**	4164±231,8**	13,9±0,8**

Table S1. Data represents mean ± SEM of distance and velocity time in Open Field Tets (OFT) during the 5 min test period (n = 11-13 rats per group). **p < 0.01 versus SHAM according to T-student.

Table S2.

	Dorsal Hippocampus				Prefrontal Cortex			
	5HT1A	Rab5	BDNF	TRKb	5HT1A	Homer1A	GalR1	GalR2
**SHAM SS+aCSF**	1.20 ±0.1	0.97±0.1	2.53±0.6	1.30±0.2	0.94±0.1	2.27±0.4	1.95±1.1	1.21±0.1
**OBX SS+aCSF**	2.17±0.2**	1.35±0.1*	9.22±1.4**	2.77±0.2***	1.4±0.1**	0.93±0.1**	2.21±1.1	0.96±0.2

Table S2. Effects between SHAM and OBX rats in the expression of different receptors 5HT1A, Rab5, BDNF and TRKb in the Dorsal Hippocampus, and 5ht1A, Homer1A, GalR1 and GalR2 in Prefrontal Cortex in rats (n=4-5 animals per group). $^*p<0.05$, $^{**}p < 0.01$ and $^{***}p < 0.001$ versus SHAM SS+aCSF according to T-student.

Table S3. Effects between SHAM and OBX rats in the FST and TST.

	Forced Swimming Test		Tail Suspension Test
	**Immobility Time**	**Swimming Time**	**Immobility Time**
**SHAM**	87,1±9,9	128,6±13,6	177,3±13,1
**OBX**	95,1±9,3	121,2±12,5	174,8±7,6

Table S3. Data represents mean ± SEM of immobility and swimming time in Forced Swimming Test (FST) during the 5 minutes test period and the immobility time in Tail Suspension Test (TST) during 6 minute test period. There are no significant differences according to T-student test.

Table S4. Effects of the administration of siRNA 5HT1A versus delivery media with differents treatments in FST

	Treatment	Immobility Time	Swimming Time
**OBX D.M.**	**SS**	85,9±12,3	168,6±13,7
**OBX siRNA 5HT1A**	**SS**	83,7±13,7	171±13,1
**OBX siRNA 5HT1A**	**GAL(1-15)(1nmol)**	97,4±12,6	155,7±10,9

Table S4. Data represents mean ± SEM of immobility and swimming time in Forced Swiming Test (FST) during the 5 min test period (n = 8-13 rats per group). There are no significant differences according to one way ANOVA followed by Fisher Multiple Comparison Test.

Figure S1.

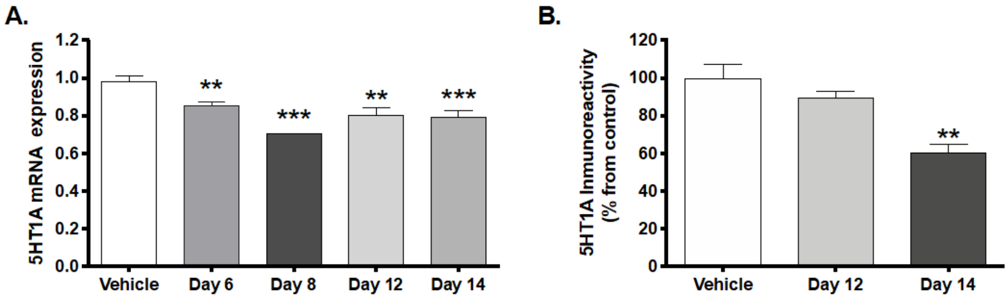

Figure S1. **A.** Expression levels of 5HT1A receptor mRNA in Hippocampus at different days obtained after the icv injection of Accell Smartpool siRNA for 5HT1A. The qPCR results were normalized to the expression levels of GAPDH and expressed as arbitrary units. ***p*<0.01 and ****p*<0.001 vs Vehicle group according to a one-way analysis of variance (ANOVA) followed by Fisher's least significance difference test. **B.** Time course of 5HT1A protein expression in Dorsal Raphe (DR) nucleus following a icv single injection of siRNA 5HT1A or vehicle. 5HT1A immunoreactivity was determined after immunofluorescent labeling. Vertical bars represent mean ± SEM of percentage of change from respective controls. **p<001 vs vehicle group according to a one-way analysis of variance (ANOVA) followed by Fisher's least significance difference test.
